# Impact of morbidity on care need increase and mortality in nursing homes: a retrospective longitudinal study using administrative claims data

**DOI:** 10.1186/s12877-020-01847-7

**Published:** 2020-10-31

**Authors:** Katrin C. Reber, Ivonne Lindlbauer, Claudia Schulz, Kilian Rapp, Hans-Helmut König

**Affiliations:** 1 Department of Health Economics and Health Services Research, Hamburg Center for Health Economics, University Medical Center Hamburg-Eppendorf, Martinistr. 52, 20246 Hamburg, Germany; 2 Department of Clinical Gerontology, Robert-Bosch-Hospital, Auerbachstr. 110, 70376 Stuttgart, Germany

**Keywords:** Care need, Competing risk, Long-term care, Mortality, Nursing home

## Abstract

**Background:**

A growing number of older people are care dependent and live in nursing homes, which accounts for the majority of long-term-care spending. Specific medical conditions and resident characteristics may serve as risk factors predicting negative health outcomes. We investigated the association between the risk of increasing care need and chronic medical conditions among nursing home residents, allowing for the competing risk of mortality.

**Methods:**

In this retrospective longitudinal study based on health insurance claims data, we investigated 20,485 older adults (≥65 years) admitted to German nursing homes between April 2007 and March 2014 with care need level 1 or 2 (according to the three level classification of the German long-term care insurance). This classification is based on required daily time needed for assistance. The outcome was care level change. Medical conditions were determined according to 31 Charlson and Elixhauser conditions. Competing risks analyses were applied to identify chronic medical conditions associated with risk of care level change and mortality.

**Results:**

The probability for care level change and mortality acted in opposite directions. Dementia was associated with increased probability of care level change compared to other conditions. Patients who had cancer, myocardial infarction, congestive heart failure, cardiac arrhythmias, renal failure, chronic pulmonary disease, weight loss, or recent hospitalization were more likely to die, as well as residents with paralysis and obesity when admitted with care level 2.

**Conclusion:**

This paper identified risk groups of nursing home residents which are particularly prone to increasing care need or mortality. This enables focusing on these risk group to offer prevention or special treatment. Moreover, residents seemed to follow specific trajectories depending on their medical conditions. Some were more prone to increased care need while others had a high risk of mortality instead. Several conditions were neither related to increased care need nor mortality, e.g., valvular, cerebrovascular or liver disease, peripheral vascular disorder, blood loss anemia, depression, drug abuse and psychosis. Knowledge of functional status trajectories of residents over time after nursing home admission can help decision-makers when planning and preparing future care provision strategies (e.g., planning of staffing, physical equipment and financial resources).

## Background

Population aging is likely to be accompanied by an increasing demand for care as a larger number of older people with chronic illness, disabilities and severe functional limitations will be expected. According to recent model calculations, the number of dependent older adults with need for care will increase from 101 million in 2010 to 277 million in 2050 globally [1]. These projections suggest that the number of people needing long-term care in institutional settings (e.g., nursing homes) will rise considerably. In Germany, the percentage of institutionalized adults aged 65 years and older increased from 3.8% in 2010 to 4.1% in 2015 [2]. Of those who qualify for the benefits of the long-term care insurance, 23% between 65 and 80 years of age live in an institution, whereas the number is about one third for those aged 80 or older [3].

The older population partly lives in rural areas where, for a long time, family members have often taken on caregiving roles. However, living arrangements among older people and their families have been changing considerably over the past years. Due to the continuing outmigration of young people to urban areas spatial proximity to children (and other kin) decreases, and other options of caregiving services are required [4]. As a consequence, demand for nursing home care is likely to stay high or grow even further [5].

Functional decline, disability, and frailty entailed by advanced age are prevalent in the nursing home population. Regular monitoring of functional status after nursing home admission is regarded as a core measure informing the type and intensity of care needed. Although data are available on the functional status trajectory of community-dwelling older people, less is known regarding nursing home residents [6]. The functional status indicates frailty and dependency of nursing home residents on assistance to perform activities of daily living [7] (ADL). Care dependents may have to deal with impaired mobility, restricted ability to be alone, and reduced quality of life [8]. The care need of nursing home residents is determined by chronic diseases [9], which implies functional status as a proxy for the overall health status.

Moreover, knowledge about functional trajectories of nursing home residents and their care needs is important, as this requires anticipatory planning of care and management of resources [10]. Long-term care in institutional settings is expensive and accounts for the majority of long-term care spending [11, 12]. Projections reveal a continuing growth of public long-term care expenditures over the next decades [11, 13]. On average, OECD countries spent about 1.5% of their GDP on long-term care. In Germany, public expenditures on long-term care in general accounted for 1.4% of GDP in 2013, with expenditures on residential long-term care amounting to approx. 0.9–1% of GDP [14, 15].

Several medical conditions and resident characteristics have been discussed to be potential risk factors predicting negative health outcomes like a worsened functional performance or mortality [16, 17]. Such a deterioration of health status may be associated with a change in the care need (i.e., transition to a higher care need level). Identification of and information about the specific risk profile of older adults admitted to nursing homes is therefore crucial for health policy decision makers, nursing homes and families in order to allocate (financial) resources and to meet future care expenses.

In Germany, care recipients are compulsorily classified according to their care need by the long-term care insurance. To claim benefits from the long-term care insurance, people must need a daily minimum of assistance with basic ADL (washing, eating, toileting, or dressing) and instrumental ADL (preparing meals, shopping, cleaning, using the telephone, or moving within the community). The verification and assessment of care needs is performed by the medical service of the health insurance funds according to a standardized procedure. Depending on the amount of care required recipients are classified into one of three levels: care level 1 recipients need at least 45 min of basic care a day and at least 90 min in total of general help (ADL/instrumental ADL combined). Care level 2 and 3 recipients require assistance for at least 180 and 300 min a day for general help, of which a minimum of 120 and 240 min is dedicated to basic ADLs, respectively [18]. The categorization in one of the three levels of care can therefore be regarded as a surrogate of disability [19].

The aim of this study was to examine the association of a wide range of chronic medical conditions with risk of increasing care need in terms of care level change (to a higher level) among nursing home residents. Given that nursing home residents are highly susceptible to mortality [20] which may prevent them from care level change, mortality may alter the chance of care level change to occur [21]. Ignoring mortality may thus not give accurate estimates of risk of care level change [22, 23]. Therefore, we applied an estimation method that explicitly takes into account the competing risk of mortality.

## Methods

### Data sources and study population

Routinely collected health insurance claims data on age, sex, in- and outpatient diagnoses, hospitalization stay, status of care level, nursing home residency, and date of death, if applicable, were used. All data were provided by the social insurance for agriculture, forestry, and horticulture, which mainly covers agriculturists and their families.

Within the German long-term care system, the need for care is described by three care levels that take account for the support and intensity required in ADL. Care level 1 is intended for mild dependency, care level 2 for moderate dependency and care level 3 reflects severe dependency. We considered individuals admitted to a nursing home with care level 1 or 2 between April 2007 and March 2014 who were 65 years or older at admission. Our outcome of interest was care level change to a higher level. Therefore, we excluded individuals assigned to care level 3 prior to nursing home admission because they could not move to a higher care level.

### Outcome variable

The primary outcome measure was time from nursing home admission to care level change (to a higher care level). Mortality was treated as competing event. Individuals were followed from the date of nursing home admission (baseline). For risk estimation, the time to a care level change was calculated from baseline to either date of care level change (event of interest) or mortality (competing event). Individuals alive without care level change were censored, as were those who left nursing homes.

### Study variables

Baseline characteristics recorded at the time of nursing home admission included age and sex. Age was classified in 10-year age groups in order to provide a sufficient number of observations per age group. We also observed whether an individual was hospitalized within 60 days prior to nursing home admission because it has been demonstrated that such a proximal event can have deleterious effects on health outcomes and may be predictive of (further) functional decline and dependence in older adults [24, 25]. For each individual the presence or absence of several medical conditions (at baseline) was determined. This selection of medical conditions was based on a previously described combination of conditions from the Charlson Index [26] and the Elixhauser comorbidity system [27, 28]. Inpatient and outpatient diagnoses based on the *International Classification of Diseases, 10th revision* (*ICD-10*) recorded up to 12 months prior to nursing home admission (plus an additional 14-days grace period after nursing home admission) were considered in the calculations. A final set of 31 medical conditions was included in the analyses. However, the prevalence of HIV in our sample was negligible and therefore removed.

### Statistical analysis

Descriptive statistics were used to summarize the baseline characteristics of our study sample. The study variables at baseline were compared according to care level at admission. Cumulative incidence functions were used to depict the absolute risk of the event of interest, care level change, and the competing event, mortality, over time.

For multivariate modelling we applied the subdistribution hazard model proposed by Fine and Gray [29]. Mortality during follow-up was treated as competing risk. In the Fine and Gray model, individuals who died prior to experiencing a care level change were not censored but were kept in the risk set. The assumption of proportional hazards was evaluated by visual inspection of Schoenfeld-type residuals and by including interaction terms between covariates and time. No violation of this assumption was found for the medical conditions covariates.

Models were stratified according to care level at admission and adjusted for age, sex and having been hospitalized during the 60 days prior to nursing home admission (yes/no). As health insurance claims data usually comprise a high number of observations, a *p*-value less than .01 was considered statistically significant [30]. Data analyses were performed using SAS, version 9.3 (SAS Institute, Inc., Cary, NC) and the “pshreg” macro [31].

## Results

### Sample characteristics

The study population consisted of 20,485 individuals who met our inclusion criteria, out of which 10,048 (49.1%) were admitted to a nursing home with care level 1 and 10,437 (50.9%) with care level 2. Those with care level 1 had a mean age of 83.8 (SD: 6.3) years, and 63.3% were female. Individuals admitted with care level 2 were, on average, 84.1 years (SD: 6.5) old, and 59.8% were female. In total, 7575 nursing home residents experienced an increase of care need (4962 residents changed from care level 1 to a higher care level, i.e., care level 2 or 3, and 2613 residents changed from care level 2 to care level 3), 7860 residents died, and 5050 residents were censored. The median time to care level change was 751 days (minimum 2 days, maximum 3255 days).

Of the 10,048 individuals admitted with care level 1, 56.1% had a hospital stay within 60 days before nursing home admission, and of the 10,437 individuals admitted with care level 2, the proportion was 57.8%. For individuals admitted with care level 1, most prevalent medical conditions present at baseline were hypertension (78.0%), congestive heart failure (48.2%), dementia (42.1%), cardiac arrhythmias (37.2%), and cerebrovascular disease (32.1%). Figures were similar for those admitted with care level 2, e.g. hypertension (76.6%) was most prevalent followed by dementia (50.8%), congestive heart failure (49.9%), cerebrovascular disease (41.3%), and cardiac arrhythmias (38.6%) (Table 1).

**Table 1:** Resident characteristics according to care level at admission

	Care level 1		Care level 2	
	*n* = 10,048		*n* = 10,437	
	N (%)		N (%)	
Age (in yrs) at nursing home admission, mean (SD)	83.8	(6.3)	84.1	(6.5)
Age group (at baseline)				
65- < 75 yrs	967	(9.6)	994	(9.5)
75- < 84 yrs	4444	(44.2)	4514	(43.2)
85- < 94 yrs	4371	(43.5)	4483	(43)
95+ yrs	266	(2.6)	446	(4.3)
Female	6360	(63.3)	6245	(59.8)
Follow-up				
Care level change	4962	(49.4)	2613	(25)
Mortality	2523	(25.1)	5337	(51.1)
Hospitalization stay (before baseline)				
Up to 60 days	5633	(56.1)	6034	(57.8)
Medical conditions (at baseline)				
Alcohol abuse	261	(2.6)	221	(2.1)
Any tumor	1649	(16.4)	1837	(17.6)
Blood loss anemia	113	(1.1)	117	(1.1)
Cardiac arrhythmias	3736	(37.2)	4026	(38.6)
Cerebrovascular disease	3226	(32.1)	4313	(41.3)
Chronic pulmonary disease	2454	(24.4)	2497	(23.9)
Coagulopathy	498	(5.0)	477	(4.6)
Complicated diabetes	1398	(13.9)	1506	(14.4)
Congestive heart failure	4848	(48.2)	5212	(49.9)
Deficiency anemias	822	(8.2)	846	(8.1)
Dementia	4235	(42.1)	5303	(50.8)
Depression	3209	(31.9)	3076	(29.5)
Drug abuse	103	(1.0)	89	(0.9)
Fluid and electrolyte disorders	1730	(17.2)	2284	(21.9)
Hypertension	7834	(78.0)	7996	(76.6)
Hypothyroidism	829	(8.3)	864	(8.3)
Liver disease	934	(9.3)	822	(7.9)
Metastatic cancer	397	(4.0)	576	(5.5)
Myocardial infarction	581	(5.8)	615	(5.9)
Neurodegenerative disorders	1456	(14.5)	2305	(22.1)
Obesity	1127	(11.2)	1153	(11.0)
Paralysis	573	(5.7)	1379	(13.2)
Peripheral vascular disorder	1991	(19.8)	2092	(20.0)
Psychosis	554	(5.5)	555	(5.3)
Pulmonary circulation disorders	445	(4.4)	443	(4.2)
Renal failure	2181	(21.7)	2350	(22.5)
Rheumatoid arthritis/collagen vascular diseases	773	(7.7)	792	(7.6)
Ulcer disease	344	(3.4)	392	(3.8)
Uncomplicated diabetes	3096	(30.8)	3373	(32.3)
Valvular disease	1464	(14.6)	1448	(13.9)
Weight loss	456	(4.5)	570	(5.5)

Unless otherwise indicated, values are expressed as numbers (percentages) of individuals

*yrs* years, *SD* standard deviation

**Fig. 1:**
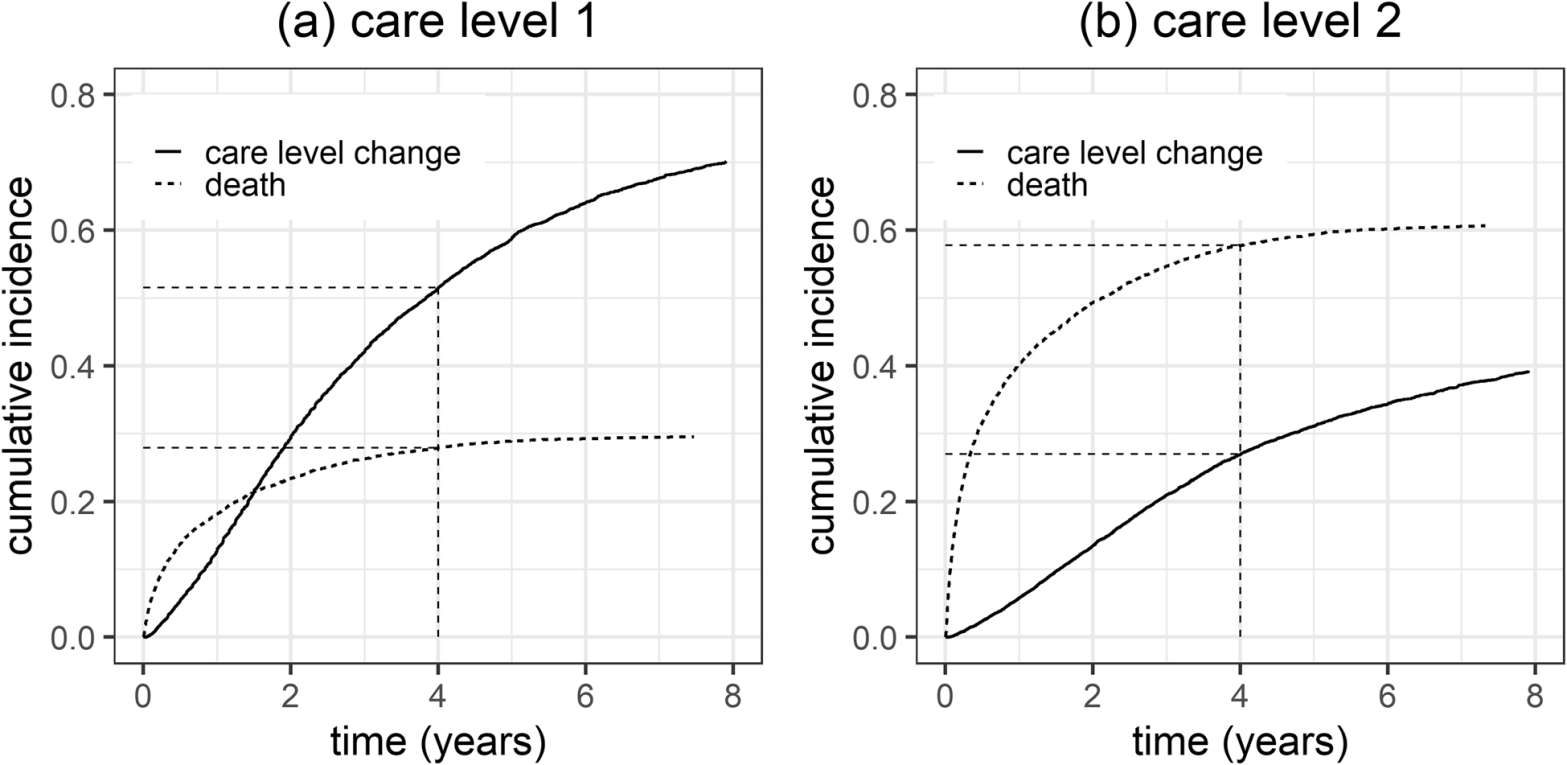
Cumulative incidence curves of care level change and mortality for residents admitted with care level 1 (**a**) and care level 2 (**b**)

Cumulative incidence curves for care need increase and mortality are shown in Fig. 1.

Fine and Gray models were fitted for care need increase and mortality. We regressed the hazard of care need increase and mortality (the competing event), respectively, on the risk factors described above. In Table 2, the estimated subdistribution hazard ratios (sHR) and their corresponding confidence intervals (CI) are shown.

**Table 2:** Results of the multivariate subdistribution hazard models for care level change and the competing event mortality, stratified by care level at admission

	Care level 1 at admission						Care level 2 at admission					
	Care level change			Mortality			Care level change			Mortality		
	sHR	95% CI	*P*-Value	sHR	95% CI	*P*-Value	sHR	95% CI	*P*-Value	sHR	95% CI	*P*-Value
Female	1.03	0.97–1.10		0.76	0.69–0.82	< 0.001	1.04	0.96–1.13		0.85	0.80–0.90	< 0.001
Age (per 10-y increase)	1.02	0.98–1.07		1.41	1.31–1.52	< 0.001	0.85	0.80–0.90	< 0.001	1.26	1.20–1.32	< 0.001
Hospitalization stay (60 days before)	0.84	0.79–0.88	< 0.001	1.48	1.36–1.61	< 0.001	0.77	0.71–0.83	< 0.001	1.43	1.35–1.52	< 0.001
Myocardial infarction	0.81	0.70–0.92	< 0.01	1.33	1.14–1.55	< 0.001	0.79	0.65–0.96	< 0.02	1.29	1.15–1.44	< 0.001
Congestive heart failure	0.93	0.87–0.98	< 0.01	1.22	1.12–1.33	< 0.001	0.83	0.77–0.90	< 0.001	1.17	1.11–1.25	< 0.001
Peripheral vascular disorder	0.97	0.90–1.05		1.08	0.98–1.19		1.01	0.91–1.12		1.04	0.97–1.11	
Cardiac arrhythmias	0.97	0.91–1.03		1.13	1.04–1.23	< 0.01	0.90	0.82–0.98	< 0.02	1.14	1.08–1.21	< 0.001
Valvular disease	0.95	0.87–1.04		1.11	1.00–1.25		0.94	0.83–1.07		1.05	0.97–1.14	
Coagulopathy	0.86	0.74–0.99	< 0.04	1.12	0.95–1.33		0.87	0.70–1.09		1.07	0.94–1.22	
Chronic pulmonary disease	0.95	0.89–1.02		1.14	1.04–1.25	< 0.01	0.93	0.84–1.02		1.11	1.04–1.18	< 0.01
Pulmonary circulation disorders	0.90	0.78–1.05		1.17	0.98–1.40		0.85	0.68–1.07		1.15	1.01–1.31	< 0.05
Rheumatoid arthritis/collagen vascular d.	0.95	0.85–1.05		0.99	0.85–1.15		0.91	0.79–1.06		1.06	0.96–1.18	
Ulcer disease	0.83	0.71–0.98	< 0.03	1.24	1.01–1.53	< 0.05	0.91	0.72–1.13		1.04	0.91–1.19	
Any tumor	0.91	0.83–0.99	< 0.04	1.44	1.30–1.60	< 0.001	0.72	0.63–0.82	< 0.001	1.50	1.39–1.62	< 0.001
Metastatic cancer	0.67	0.54–0.83	< 0.001	2.45	2.06–2.92	< 0.001	0.44	0.32–0.59	< 0.001	2.27	1.99–2.58	< 0.001
Cerebrovascular disease	1.06	1.00–1.12		0.94	0.86–1.03		0.98	0.91–1.06		1.01	0.95–1.07	
Dementia	1.43	1.36–1.51	< 0.001	0.73	0.67–0.80	< 0.001	1.74	1.60–1.89	< 0.001	0.73	0.69–0.78	< 0.001
Neurodegenerative disorders	1.10	1.02–1.18	< 0.02	0.92	0.82–1.04	0.17	1.03	0.94–1.13	0.48	0.99	0.93–1.06	0.80
Paralysis	1.04	0.92–1.17		1.01	0.84–1.22		1.00	0.89–1.13		0.88	0.80–0.96	< 0.01
Complicated diabetes	1.05	0.96–1.15		0.95	0.83–1.08		0.90	0.79–1.02		1.10	1.00–1.20	< 0.05
Uncomplicated diabetes	1.04	0.97–1.11		1.04	0.94–1.14		1.00	0.91–1.09		1.00	0.93–1.06	
Renal failure	0.97	0.90–1.05		1.19	1.08–1.31	< 0.001	0.88	0.79–0.97	< 0.02	1.16	1.08–1.24	< 0.001
Liver disease	1.06	0.96–1.17		0.99	0.86–1.14		0.90	0.77–1.05		1.05	0.94–1.16	
Obesity	1.00	0.92–1.09		0.95	0.83–1.09		1.02	0.90–1.16		0.87	0.80–0.96	< 0.01
Depression	0.96	0.91–1.02		0.95	0.87–1.04		0.98	0.90–1.06		0.94	0.89–1.00	
Hypertension	0.98	0.91–1.04		0.96	0.87–1.06		1.06	0.97–1.16		0.93	0.87–0.99	< 0.04
Hypothyroidism	0.96	0.87–1.06		0.98	0.84–1.13		1.07	0.94–1.23		0.91	0.82–1.01	
Fluid and electrolyte disorders	1.08	1.00–1.17	< 0.04	1.03	0.93–1.15		1.00	0.91–1.10		1.04	0.97–1.11	
Blood loss anemia	1.01	0.74–1.39		0.95	0.65–1.39		0.85	0.58–1.23		1.20	0.96–1.50	
Deficiency anemias	0.93	0.83–1.03		1.09	0.95–1.25		0.92	0.79–1.07		1.08	0.97–1.19	
Weight loss	0.87	0.76–1.01		1.51	1.27–1.78	< 0.001	0.85	0.70–1.04		1.38	1.23–1.55	< 0.001
Alcohol abuse	0.82	0.69–0.98	< 0.03	1.28	0.99–1.65		0.78	0.58–1.03		1.07	0.88–1.30	
Drug abuse	0.98	0.75–1.29		0.65	0.39–1.08		1.06	0.71–1.58		1.01	0.76–1.35	
Psychosis	0.93	0.84–1.05		1.00	0.83–1.21		0.95	0.81–1.11		0.88	0.78–1.00	

*sHR* subdistribution hazard ratio, *CI* confidence interval, *y* year; collagen vascular d., collagen vascular disease

### Cumulative incidence of care level change and mortality

Figure 1 provides the cumulative incidence curves for both possible outcomes, care level change and mortality, considering competing risks. The cumulative incidence of care level change by year four was 51.1% (95% CI 50.4–52.7) for individuals admitted with care level 1 (Fig. 1a) whereas 27.9% (95% CI 27.0–28.9) died. For those admitted with care level 2 (Fig. 1b) the cumulative incidence of a care level change by year four was 27.0% (95% CI 26.0–28.0). The cumulative incidence of mortality by year four approached 57.8% (95% CI 56.7–58.9).

### Results of the subdistribution hazard model for care level change

Multivariate competing risk analysis, adjusting for age, sex and prior hospitalization, showed that residents with metastatic cancer (sHR 0.67, 95% CI 0.54–0.83), myocardial infarction (sHR 0.81, 95% CI 0.70–0.92) or congestive heart failure (sHR 0.93, 95% CI 0.87–0.98) were more likely to keep their level of care need when admitted to a nursing home with care level 1 compared to residents without the specific medical condition. Residents admitted with care level 2 who had metastatic cancer (sHR 0.44, 95% CI 0.32–0.59), any tumor (sHR 0.72, 95% CI 0.63–0.82), or congestive heart failure (sHR 0.83, 95% CI 0.77–0.90) also had a decreased probability. In contrast, dementia significantly increased the probability of care need increase, regardless of a resident’s care level at admission (care level 1: sHR 1.43, 95% CI 1.36–1.51; care level 2: sHR 1.74, 95% CI 1.60–1.89). Remaining medical conditions did not achieve the threshold for statistical significance.

### Results of the subdistribution hazard model for the competing risk (mortality)

Regardless of residents’ care level upon admission, when having metastatic cancer (care level 1: sHR 2.45, 95% CI 2.06–2.92; care level 2: sHR 2.27, 95% CI 1.99–2.58), any tumor (care level 1: sHR 1.44, 95% CI 1.30–1.60; care level 2: sHR 1.50, 95% CI 1.39–1.62), myocardial infarction (care level 1: sHR 1.33, 95% CI 1.14–1.55; care level 2: sHR 1.29, 95% CI 1.15–1.44), and congestive heart failure (care level 1: sHR 1.22; 95% CI 1.12–1.33; care level 2: sHR 1.17; 95% CI 1.11–1.25), residents were more likely to die. Other conditions associated with mortality were cardiac arrhythmias, chronic pulmonary disease (COPD), renal failure, and weight loss. In contrast, residents with dementia were less likely to die when admitted with both care level 1 and 2 (care level 1: sHR 0.73; 95% CI 0.67–0.80; care level 2: sHR 0.73; 95% CI 0.69–0.78). Residents admitted with care level 2 with obesity or paralysis also showed a lower risk of mortality. Remaining medical conditions did not achieve the threshold for statistical significance (Table 2).

Furthermore, residents of older age (care level 1: sHR 1.41; 95% CI 1.31–1.52; care level 2: sHR 1.26; 95% CI 1.20–1.32) and with a hospital stay prior to nursing home admission (care level 1: sHR 1.48; 95% CI 1.36–1.61; care level 2: sHR 1.43; 95% CI 1.35–1.52) were more likely to die, and females (care level 1: sHR 0.76; 95% CI 0.69–0.82; care level 2: sHR 0.85; 95% CI 0.80–0.90) were less likely to die.

## Discussion

To analyze the association of the risk of care need increase in terms of care level change and a wide range of medical conditions among nursing home residents, we used health insurance claims data of more than 20,000 individuals admitted to a nursing home between 2007 and 2014. We considered medical conditions based on conditions included in the Charlson Index and the Elixhauser comorbidity system and designed specifically for use with administrative data [28]. As this population is particularly vulnerable to the competing risk of mortality, we employed models that explicitly allow considering this competing event.

We found that in this large nursing home population, after controlling for age, sex and hospital stay prior to nursing home admission, residents with dementia were more likely to sustain a care need increase. In contrast, residents with the chronic medical conditions cancer, congestive heart failure, and myocardial infarction were less likely to sustain a care need increase. Patients with these conditions were also found to be more likely to die, in addition to having any tumor, renal failure, COPD, and weight loss as well as paralysis and obesity when admitted with care level 2. Though our findings seem quite robust, given the large study sample, several medical conditions for which one might have expected an effect, were neither particularly related to care need increase nor mortality (e.g., diabetes, cerebrovascular disease). Also prior studies have described mixed results for e.g., diabetes or cerebrovascular disease regarding their association with survival in nursing homes [17, 32, 33]. It appeared that individuals with these conditions are less predictable as regards their functional trajectories. Possibly, the severity of the medical conditions of these individuals is more heterogeneous or there are further health-related issues which are not recorded in health insurance claims data.

Earlier research postulated that different trajectories of disability and decline exist when approaching the end of life [34, 35]. Depending on prevalent medical conditions individuals may follow specific trajectories: terminally ill individuals (e.g., with cancer) or those with organ failure (e.g., heart failure, COPD) were more independent in their ADLs than frail people or those with dementia. These individuals may more likely die than sustain a care need increase, opposite to dementia patients. Terminally ill and organ failure individuals also showed a more modest or erratic functional decline until they reached the very last months of life [34]. Our results are quite in line with these earlier findings. There seem to exist those nursing home residents with cancer or congestive heart failure that fall into the terminally ill or organ failure group and are more likely to die than to change care level, those with dementia or other neurodegenerative who deteriorate further after nursing home placement requiring a care level change, and those that fall in none of these groups showing a less predictable pattern of decline.

Specifically, our results revealed that the probability for care level change and mortality worked in opposite directions. Dementia was identified to be the strongest risk factor associated with a care level change; while cancer was shown to exert the strongest effect on the cumulative incidence of mortality. Residents with dementia (compared to those without dementia) had a higher probability of experiencing a care level change and likely to remain long-term stayers. This may derive from the fact that in nursing homes dementia patients are more likely to sustain a care need increase and less likely to die compared to residents with other diseases and, therefore, are under risk for care need increase for a longer period of time, which would make it more likely to occur. In contrast, cancer decreased the probability of a care level change. Residents with cancer (compared to those without cancer) seem to have a quicker time to mortality and this competing event will occur before being able to encounter a care level change.

In support of our results, previous studies found dementia to be a clear predictor of ADL decline impeding physical function in various durations, e.g., due to psychological symptoms or acute illness related to dementia [36–38]. Earlier studies further showed an overall decline in ADL over time in long-stay nursing home residents with cognitive impairments. These elevated levels of physical impairments in turn necessitate greater support and assistance which translates in higher care burden and eventually in higher levels of care [38]. Results from two recent follow-up studies among nursing home residents in the Netherlands and Austria showed that dementia was predictive of an increase in care dependency over time [39, 40]. Another study of nursing home residents with dementia indicated that the rate of decline in ADL functioning associated with dementia decreased over time [41]. It was further found that nursing home residents admitted with dementia had a lower risk of mortality compared to residents without dementia [42]. Moreover, some forms of dementia were associated with longer survival [42–44], however changes in care service use and the extent of care may vary with varying forms of dementia [45, 46].

The profile of adverse outcomes in residents admitted with dementia diverges from that of residents without dementia, suggesting that the two groups exhibit different disability patterns and thus care trajectories and distinct long-term care needs [35, 42]. Using the nomenclature by Gill and colleagues [35], nursing home residents with dementia showed “persistent” or “progressive” patterns of disability (i.e. constant or slightly increasing disability) whereas in residents without dementia “accelerated” or “catastrophic” disability trajectories (i.e. faster or higher increase of disability) occurred. In these latter cases functional deterioration (resulting in death) happens rapidly precluding transfer to a higher care level. It is thus important for nursing home staff, residents as well as their relatives to take this into account. For the residents this may suggest a higher degree of dependence on nursing home staff or relatives as well as a higher amount of payment for care, and for the relatives it may possibly indicate more time needed for additional informal care. Nursing homes should consider the potentially increased resource utilization when preparing and planning resources for future care in terms of time or staff needed or environmental adaptions in order to address the special needs of residents with increased care need due to e.g., dementia.

Regarding cancer, a higher risk of mortality has been previously observed in the long-term care setting [17, 33, 36]. Similar to our findings, studies also found heart failure [17, 32, 33], chronic lung disease [17, 36], and weight loss [47–49] to be important risk factors for mortality in nursing home populations.

Possibly, there may be particular medical conditions that do not act individually but synergistically with other medical conditions. Together they may affect adverse outcomes like worsening of care level or mortality. Prior studies have pointed to the importance of synergistic effects of specific disease combinations. Some combinations were suggested to contribute to a decline in functional ability or higher mortality risk than one may expect when considering the effects of these diseases separately (e.g., arthritis and heart disease, depression and cognitive impairment) [17, 50, 51]. However, a study of mortality in a large population of institutionalized older people found only one (out of 36) disease combination (i.e., dementia and stroke) that emerged consistently as risk factor in their mortality risk prediction models [17]. The authors argued that disease severity rather than the mere presence of the disease could have been a more sensitive measure when analyzing synergistic effects. Alternatively, there could be an interaction between medical conditions prevalent upon nursing home admission and medical conditions that develop during the nursing home stay that affect adverse health outcomes [51]. We addressed this issue by investigating a large number of medical conditions and applying an appropriate model which considered changes over time.

Major strengths of our study are the large number of individuals admitted to a nursing home, a relatively long observation period that allowed us to examine long-term outcomes associated with several disease-related risk factors present upon nursing home admission, and an objective and well-established measure of medical conditions. We employed competing risk regression to study the association between several medical conditions and the risk of care need increase. We preferred this approach over a standard survival model because in nursing home populations, mortality may be a common competing risk, and ignoring such competing risks may lead to biased results [52]. In the presence of competing events, the traditional Kaplan-Meier method is inadequate to estimate the probability of the occurrence of an event (i.e., care level change or mortality) as it treats the competing event as a censored observation. Kaplan-Meier estimates the cumulative incidence in the absence of competing risks, which may be appropriate for censoring by loss to follow-up but less suitable for censoring by e.g., mortality. Consequently, Kaplan-Meier results in biases in the estimation of the probability of the event of interest [53]. In competing risk situations, the cumulative incidence function is therefore considered as more appropriate as it takes competing events into account when estimating the incidence, e.g., individuals who experience the competing event (mortality) first can no longer move on to experience the event of interest (care level change). We further chose the Fine and Gray model over the cause-specific hazard model as our primary interest was in predictive modelling. An appealing feature of the Fine and Gray model is that it allows estimating the effect of covariates on the cumulative incidence function of the event of interest [54]. When the primary interest is in risk prediction and prognosis, modelling absolute risk of the outcome, say care level change, rather than the instantaneous rate of the outcome in those who are event free has been suggested [20, 22]. As our motivation was to predict the risk of care level change, we preferred to use the Fine and Gray model that directly offers estimated probabilities of the event of interest occurring over time, given an individual’s risk factors.

Our study has some limitations that should be considered when interpreting our results. First, we only had information available from administrative claims databases. Factors such as nursing staff quality or facility characteristics that might have affected our outcome could therefore not be controlled for. Second, identification of individuals’ medical conditions was based on inpatient and outpatient diagnoses. While diagnostic misclassification should be low in the inpatient setting, the outpatient setting is prone to inaccurate diagnosis coding. Therefore, it is possible that some medical conditions (particularly those mainly diagnosed in an outpatient setting) were not or incorrectly specified. Third, we analyzed data between April 2007 and March 2014, which are already some years old. However, we intended to investigate care level change of residents during their nursing home stay, which requires a long follow-up. Furthermore, the German classification of care need was changed from three care levels to five care degrees in January 2017. In order to avoid biases, we refrained from investigating more recent data. Otherwise, no substantial changes to the regulation of nursing homes care were made. Therefore, the results of our analysis should still be valid and relevant. Fourth, we used data from the social insurance for agriculture, forestry, and horticulture. Thus, the results may not be representative for the German nursing home population as a whole. However, it may be considered representative of the nursing home population previously active in agriculture, horticulture or forestry who was therefore compulsorily insured at this social insurance. Due to the demographic shifts and rural-urban migration, this population will become increasingly important as rural areas remain disproportionally older. However, direct comparison of our findings with results from the literature is complicated due to different follow-up periods, different methodological approaches, different settings or care systems. Moreover, institutionalization and nursing home care depend on access to and availability of care facilities as well as on financial and human resources which may vary from country to country. This will restrict comparability and generalizations across health care systems.

## Conclusion

Our competing risk analysis based on administrative claims data identified risk groups of nursing home residents which are particularly prone to increasing care need or mortality. Dementia was an important factor for care need increase regardless of a resident’s care level at admission. Nursing home residents with dementia were more likely to experience a care need increase and more likely to live longer in a nursing home than residents without dementia. Thus, residents with dementia may remain long-stay residents in nursing homes compared to those without dementia. In contrast, residents with other prevalent diseases like metastatic cancer, any tumor, renal disease, COPD, myocardial infarction or congestive heart failure had a lower risk of care need increase, but a higher mortality risk compared to residents without these diseases. Several conditions were neither related to increased care need nor mortality, suggesting a less predictable pattern. These conditions were valvular, cerebrovascular or liver disease, peripheral vascular disorder, rheumatoid arthritis/collagen vascular disease, hypothyroidism, blood loss anemia, deficiency anemias, depression, psychosis, and drug abuse.

A better understanding of functional status trajectories over time after nursing home admission may help caregivers and clinicians to focus on these risk groups with regard to prevention or special treatment. Furthermore, it may help decision-makers when planning and preparing future care provision strategies (e.g., planning of staffing, physical equipment and financial resources).

## Data Availability

We declare that the data are owned by the German social insurance SVLFG. Since public deposition of the data would breach ethical and legal compliance, data are only available upon formal request from the SVLFG. To request the data please contact the institutional body of the SVLFG directly (poststelle@svlfg.de-mail.de). In order to fulfill the legal requirements to obtain that kind of data, researchers must conclude a contract with the SVLFG regarding data access. The licensee is permitted to use the data for the purpose of the research proposal only. Licensees are not allowed to pass the data to a third party, or to create Software or data bases with the exception of scientific publications. Moreover, the study has to be approved by the data protection officer both at the SVLFG and the research institute.

## Abbreviations

ADL: Activities of daily living
CI: Confidence interval
COPD: Chronic obstructive pulmonary disease
GDP: Gross domestic product
HIV: Human immunodeficiency virus
ICD-10: International Classification of Diseases, 10th revision
OECD: Organisation for Economic Co-operation and Development
SD: Standard deviation
SHR: Subdistribution hazard ratios
yrs: Years
y: Year
collagen vascular d.: Collagen vascular disease
